# Case Report: Wide Spectrum of Manifestations of Ligase IV Deficiency: Report of 3 Cases

**DOI:** 10.3389/fimmu.2022.869728

**Published:** 2022-05-03

**Authors:** Ana Costa e Castro, Raquel Maia, Sara Batalha, João Parente Freixo, Catarina Martins, Conceição Neves, Ana Isabel Cordeiro, João Farela Neves

**Affiliations:** ^1^Pediatrics, Hospital Dona Estefânia, Centro Hospitalar Universitário Lisboa Central (CHULC), Lisboa, Portugal; ^2^Pediatric Hematology Unit, Hospital Dona Estefânia, Centro Hospitalar Universitário Lisboa Central (CHULC), Lisboa, Portugal; ^3^Centro de Genética Preditiva e Preventiva, Instituto de Biologia Molecular e Celular, Instituto de Investigacão e Inovacaão em Saúde, Porto, Portugal; ^4^Comprehensive Health Research Centre (CHRC), NOVA Medical School, Nova University of Lisbon, Lisbon, Portugal; ^5^Chronic Diseases Research Centre (CEDOC), NOVA Medical School, Nova University of Lisbon, Lisbon, Portugal; ^6^Primary Immunodeficiencies Unit, Hospital Dona Estefânia, Centro Hospitalar Universitário Lisboa Central, Lisboa, Portugal

**Keywords:** ligase iv, immunodeficiency, bone marrow failure, case report, hypopigmentation, lymphopenia

## Abstract

DNA ligase IV deficiency is a rare autosomal recessive disorder associated with impaired DNA repair mechanisms. Most patients with DNA repair defects present with neurologic deficits, combined immunodeficiency, bone marrow failure, and/or hematologic neoplasia. We present 3 unrelated cases of ligase IV deficiency with different clinical presentations. Patient 1 presented at the age of 5 with bone marrow failure, dysmorphic features, and T and B lymphopenia. A compound heterozygous variant *L19W/K635fs* in the *LIG4* gene was identified. Patient 2 presented at the age of 16 with recurrent infections. He had agammaglobulinemia and absent B cells. A homozygous *R278H* in the *LIG4* gene was identified. Patient 3 was referred for vitiligo and B-cell lymphopenia (low class-switched B cells) and hypogammaglobulinemia. Homozygous *R278H* in *LIG4* was also identified. In the last few years, the spectrum of clinical manifestations caused by ligase IV deficiency has widened, making it very difficult to establish an accurate clinical diagnosis. The use of NGS allows a proper diagnosis and provides a better prognosis and adequate family counseling.

## Introduction

Every day, our body cells are exposed to 10–50 DNA double-strand breaks (DNA-dsb) resulting from intracellular (DNA replication, meiosis) and extracellular events (reactive oxygen species, drugs, ionizing radiation). In order to prevent DNA damage, several proteins are involved in recognition and correction of DNA-dsb (1–3).

Two pathways are important to resolve the damage and maintain genome stability following DNA-dsb: homologous recombination and non-homologous end-joining (NHEJ) (3, 4). The first one requires an extensive sequence homology between the broken DNA and a donor DNA molecule and entails a templated DNA synthesis as a key step in the repair process. In mammalian cells, the classical repair mechanism is NHEJ, a rapid, high-capacity pathway that joins two DNA ends with minimal reference to the DNA sequence (5).

A number of proteins are involved in the NHEJ repair pathway and are conserved throughout evolution, indicating their critical role in maintaining genomic stability (4).

DNA repair machinery proteins also have a crucial role in the adaptative immune system through the generation of T- and B-cell receptors and immunoglobulins (2). In this process, the variable (V), diversity (D), and joining (J) elements of the T-cell receptor and immunoglobulin genes are targeted by recombinase activating gene (RAG)1 and RAG2 proteins that introduce DNA-dsb. The resulting DNA ends are recognized and resolved by the proteins of the NHEJ pathway: DNA ends are stabilized by a *Ku70/Ku80* heterodimer, allowing the recruitment of the DNA-PK catalytic subunit (DNA-PKcs) and activation of Artemis, which opens the hairpin-sealed DNA coding ends; finally, the coding ends are ligated by DNA ligase IV and its cofactors XRCC4 and XLF (6–8).

Defects in a number of these proteins may lead to mutagenesis and premature cell death by apoptosis (1). Because the *V(D)J* recombination process is mandatory for T and B lymphocyte development, defects in the NHEJ pathway may lead to the dysfunction of T and B cells, resulting in combined immunodeficiency (6). Most of these patients share a syndrome of neurologic deficits, combined immunodeficiency, bone marrow failure, and/or hematologic neoplasia (2).

DNA ligase IV, encoded in the *LIG4* gene, is a component of the NHEJ pathway essential for the development of a healthy immune system as well as for the protection of genome integrity (9). The *LIG4* deficiency syndrome is an extremely rare autosomal recessive disorder associated with impaired DNA-dsb repair mechanisms. The knockout models of the gene *LIG4* resulted in embryological lethality, suggesting that a null mutation might also be nonviable in human beings (6, 8, 9). This is why known human mutations are hypomorphic, leading to significant impaired NHEJ but still maintaining some activity (1, 2).

Since the first description in 1990 (10, 11), *LIG4*-deficient patients have classically been described as microcephalic with facial dysmorphism, developmental delay, growth failure, a severely compromised immune system, bone marrow failure, and a predisposition to lymphoid malignancy. However, more recently, it has been shown that the *LIG4* deficiency syndrome presents with a wide range of phenotypes (1, 2, 9). Clinical manifestations result from mutagenesis and apoptosis due to a progressive accumulation of DNA-dsb in fetal neurons and hematopoietic pluripotent stem cells (1).

Herein, we present 3 unrelated cases of ligase IV deficiency with varied clinical presentations ranging from characteristic neurodevelopment and hematologic features to asymptomatic lymphopenia and depigmented areas of the skin.

## Case Reports

**Figure 1:**
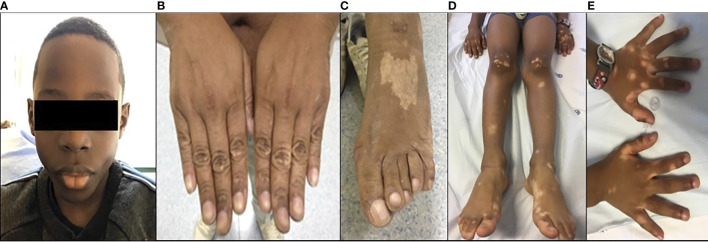
Clinical features: **(A)** microcephaly in P1; **(B, C)** hypopigmented skin lesions in P2; and **(D, E)** hypopigmented skin lesion in P3.

Patient 1 (P1) is the third male child of unrelated parents, born in Angola. Pregnancy was complicated by intrauterine growth restriction confirmed by an ultrasound scan, and he was born at 36 weeks of gestation with low birth weight. He had recurrent wheezing but did not show recurrent infections. At the age of 3, he presented thrombocytopenia and mild anemia but microcephaly without facial dysmorphic features was noted (**Figure 1A**). He also presented a partial syndactyly of the toes, as well as severe growth impairment and neurodevelopment delay. At the age of 5, he returned to our attention because of bone marrow failure with transfusion dependence, requiring regular platelet and blood transfusions (**Table 1**).

**Table 1:** Immunologic study results and clinical features from all 3 patients.

	P1	P2	P3	Reference
IgG (g/L)	18.40	**3.18**	9.74	P1/3: 5.93-17.30; P2: 7-16;
IgA (g/L)	1.94	**<0.23**	**<0.26**	P1/3: 0.33-3.60; P2: 0.7-0.4
IgM (g/L)	0.67	**<0.17**	**0.45**	P1/3: 0.55-2.1; P2: 0.4-2.3
Lymphocytes (/µl)	**750**	1,260	1,395	P1/3: 1,237-6,856; P2: 877-4,792
Lymphocyte subsets (/µl)				
B cells	**36**	**0**	**11.8**	P1/3: 157-1,637
Pregerminal	**14.7**	–	**9.5**	P1/3: 70.4-1,348
Postgerminal	**21.3**	–	**2.3**	P1/3: 35-491
Unswitched	**5.1**	–	**0.65**	P1/3: 18-333
Switched	14.1	–	**1.6**	P1/3: 14-199
T cells	**626**	961	887	P1/3: 852-5,333; P2: 611-3,477
CD4+CD8-	**276**	501	599	P1/3: 516-3,448; P2: 361-1,900
Naive	**102**	**13.5**	**43.3**	P1/3: 264-2,901; P2: 89-1,484
Central memory	160	413	374	P1/3: 152-802; P2: 121-885
Effector memory	13.1	73.9	170	P1/3: 9-222
Effector TD	0.25		12	P1/3: 0-79.3
CD8+CD4-	284	419	206	P1/3: 188-1,805; P2: 160-1,189
Naive	191	**21.3**	**25.8**	P1/3: 94-1,130; P2: 37-986
Central memory	49.5	119	65.3	P1/3: 26-456; P2: 54-427
Effector memory	11.8	2,2	12.6	P1/3: 0-151; P2: 2-515
EffectorTD CD27+d	12	56.9	45.1	P1/3: 0-385; P2: 0-144
EffectorTD CD27-	19.6	219	57.1	P1/3: 1-735; P2: 1-284
T – TCRgd+	54.9	41.7	73.7	P1/3: 44-748; P2 11-470
T – TCRgd-	9.5		8.7	P1/3: 3-104
NK cells	**88.3**	299	496	P/31: 106-1,759; P2: 81-1,021
**Clinical Features**				
Genetics (LIG4)	L19W/ K635	R278H R278H	R278H R278H	
Age at initial symptoms (years)	3	16	6	
Age at diagnosis (years)	5	21	7	
Developmental delay	No	No	No	
Recurrent infections	No	**Yes**	No	
Skin involvement	No	**Yes**	**Yes**	
Microcephaly	**Yes**	No	No	
Bone marrow failure	**Yes**	No	No	

Bold text signify values outside the reference ranges.

Bone-marrow biopsy and aspiration showed low cellularity and a left shift. Peripheral blood flow cytometry immunophenotyping revealed T, B and NK lymphopenia with preserved proliferations and immunoglobulin levels (**Table 1**).

The clinical presentation, in particular combined immunodeficiency and microcephaly, was suggestive of a DNA repair defect. The analysis of a whole exome sequencing-based 455 gene virtual panel, specifically designed for primary immunodeficiencies, revealed a compound heterozygosity in the *LIG4* gene (*L19W/K635fster*). The known pathogenic *K635fster* variant was inherited from the mother, who did not carry the L19W variant. Unfortunately, the father was unavailable for genetic testing, but the patient’s healthy sister was heterozygous for the L19W variant, allowing the conclusion that the L19W was inherited from the father.

The *L19W* mutation is unique to this patient: it was never found in humans, e.g., it is absent from more than 120,000 subjects in the gnomAD database and predicted to be pathogenic by all bioinformatic tools (CADD score 25; Mutation taster-Disease causing; Polyphen and SIFT-Damaging). Like the previously described A3V and T9I variants, it falls on the N-terminus of the protein, where the DNA-binding domain is located, and is expected to moderately impair the catalytic function of the protein. Nowadays, the patient is transfusion dependent while waiting for hematopoietic stem cell transplantation (HSCT).

Patient 2 (P2) is also an African boy who presented recurrent perianal abscesses and suppurative hidradenitis that required chirurgical attention, starting at the age of 16.

Five years later, he was admitted for pneumonia with empyema with positive blood cultures for type b *Haemophilus influenzae.* Chronic changes in the lung structure with bronchiectasis were identified on the CT scan. On physical examination, he had no microcephaly or dysmorphic features and had normal physical and mental development. The physical examination revealed hypopigmented lesions on extremities (**Figures 1B, C**). The laboratory workup showed a normal complete blood count but marked hypogammaglobulinemia (very reduced levels of serum IgG and undetectable serum levels of IgA and IgM). Immunological studies revealed absent B cells, a diminished proportion of naïve CD4^+^ and CD8^+^ T cells, and impaired T-cell proliferation to mitogen stimulation. There was no clinical or laboratory evidence of autoimmunity (**Table 1**).

Whole exome sequencing (WES) revealed a homozygous missense *R278H* mutation, inherited from his healthy parents. *R278H* was previously reported to cause *LIG4* deficiency. The patient was started on subcutaneous immunoglobulin replacement therapy and has been free of severe infections since then.

Patient 3 (P3) is a 7-year-old boy from African-origin nonconsanguineous parents that presented depigmented macules and patches with acrofacial and genital distribution since the age of 6 (**Figures 1D, E**), as well as persistent lymphopenia and hypogammaglobulinemia. The lymphocyte subpopulations further revealed persistent naive T-cell lymphopenia, as well as B-cell lymphopenia with reduced class-switched memory B cells (**Table 1**). Moreover, his past medical history was uneventful. The next-generation sequencing (NGS) PID 455 gene panel revealed the same homozygous missense *R278H* mutation in the *LIG4* gene. Today, he remains asymptomatic without treatment.

## Discussion

Most ligase IV-deficient patients reported in the literature are microcephalic children with growth retardation, beginning with an *in utero*, typical facial appearance (beak-like nose, prominent midface, receding forehead, and micrognathia) with developmental and mental delay, radiosensitivity, variable immunodeficiency, and pancytopenia (1, 9, 12). However, today, approximately 86 cases have been reported with heterogenous phenotypes, which have been reviewed in detail by Staines et al. (2, 4, 9, 13–17)

This heterogeneity in disease severity is probably linked to the amount of residual activity of DNA ligase IV and correlated with the type and position of mutations. The *LIG4* gene contains two exons, with exon 2 encoding the 911-amino acid protein. In general, biallelic truncating *LIG4* mutations are associated with a more severe phenotype than the compound-heterozygous missense and truncating variants. Furthermore, severity is correlated with the position of mutation with early truncating mutations, resulting in severe growth retardation and severe combined immunodeficiency (SCID), whereas late truncation mutations are associated with milder symptoms. However, patients with similar genotype may display variable phenotypes suggesting that additional factors may influence manifestations and severity of *LIG4* syndrome (9, 18).

According to the 2019 Update on the Classification from the International Union of Immunological Societies Expert Committee, *LIG4* syndrome is a type of SCID (19). Yet, varying degrees of susceptibility to infection, hypogammaglobulinemia and combined T and B lymphopenia have been described and most patients with this syndrome do not fulfill the criteria of SCID (2, 6, 14, 15, 20).

Flow cytometric analysis revealed CD4+ naïve and B-cell lymphopenia in all 3 patients. The depletion of naïve T cells and increased proportion of memory T cells may reflect the compensatory mechanisms of homeostatic proliferation at the expense of compromised ability to resist infections (6). Only P1 presented with typical T and B lymphopenia. NK cells may be normal or decreased in ligase IV-deficient patients (13). He had preserved serum immunoglobulin levels, likely due to the ability of the remaining B cells to develop into plasma cells and produce antibodies (13). P2 and P3 presented with an unusual phenotype: recurrent infections (P2)/autoimmunity (P3) with hypogammaglobulinemia and B-cell lymphopenia in the absence of microcephaly, dysmorphic features, developmental delay, or hematologic manifestations, resembling common variable immunodeficiency disease (CVID).

Pancytopenia, a strongly discriminative diagnostic feature of ligase IV deficiency, was only present in P1. The extent of exposure to endogenous or exogenous triggers could contribute to phenotypic variability, even in patients sharing the same genetic defects (6).

Asymptomatic and mildly symptomatic patients are also at risk of late-onset aplastic anemia and malignancy because of continuing accumulation of dsDNA breaks (2).

Short telomere length in white blood cells has also been found in LIG4 patients. So, the differential diagnosis of patients with the typical *LIG4* syndrome (P1) includes diseases such as Fanconi anemia or dyskeratosis congenita, which can also present with dysmorphic features, bone marrow failure, and radiosensitivity (16, 21, 22).

The additional reported features of ligase IV deficiency include skin conditions such as photosensitivity, psoriasis, eczema, hypopigmentation, and extensive plantar warts (1, 23). P2 and P3 presented with hypopigmentation similar to previous reports (**Figures 1B–E**) (24).

Our genetic findings were also consistent with the literature. P1, the one with the most severe phenotype, was compound heterozygous for the missense and truncating variants of the *LIG4* gene (*L19W/K635fster*), while P2 and P3 were homozygous for a missense mutation (*R278H*) (**Table 2**).

**Table 2:** Genetic results from all 3 patients.

Patients	P1	P2	P3
Zygosity	Compound heterozygous	Homozygous	Homozygous
Mutation	c.56T>G c.1904delA	c.833G>A	c.833G>A
Amino acid	p.L19W p.K635fs	p.R278H	p.R278H

With the known *K635fster* pathogenic mutation, DNA ligase IV lacks the C-terminal region responsible for XRCC4 binding and *LIG4* stability and activity; therefore, this might be a null mutation or have very low residual activity. These patients present growth retardation beginning *in utero*, syndactyly, and developmental delay but bone marrow failure was not described before in patients with K635fster mutation (12, 20). The unique *L19W* missense variant identified in heterozygosity in this patient has not been previously reported.

The *LIG4 R278H* mutation (P2 and P3) was the first *LIG4* mutation reported in humans. It is located in the conserved motif close to the active center and impairs ATP binding. As a typical hypomorphic mutation, it does not affect protein expression but reduces DNA ligase IV adenylation and ligase activity to approximately 5%–10% of wild-type protein (11, 14, 23, 25). This mutation may allow normal development, and patients harboring this variant are not expected to display overt immunodeficiency despite pronounced radiation sensitivity (25). The residual DNA ligase IV activity is probably sufficient for V(D)J recombination but does not suffice for the efficient repair of radiation-induced DNA-dsb (11).

P2 and P3 share the same genotype, and both had hypopigmented lesions, B-cell lymphopenia, and hypogammaglobulinemia. Interestingly, the immunological impairment observed in P2 was more severe than in P3. Moreover, P2 had recurrent infections while P3 did not. This could be explained by the younger age of the patient of P3, as P2 developed these symptoms later in life. Nevertheless, Girard et al. (2004) demonstrated that polymorphisms in DNA ligase IV can impact upon function, particularly when coupled with the *R278H* mutational change, and this may also underlie the difference in clinical severity between *LIG4* syndrome patients (26). Interestingly, previously described patients with the R278H mutation have been reported to either present recurrent infections starting at the age of 6, or leukemia as the first manifestation of the disease at the age of 14 (2).

The treatment of ligase IV deficiency is supportive. It aims to prevent life-threatening infections by administering antibiotic chemoprophylaxis, immunoglobulin substitution, transfusion support on a regular basis, and the avoidance of unnecessary exposure to ionizing radiation (1). Antibiotic prophylaxis was started in P1 and immunoglobulin substitution in P2. Hematopoietic stem cell transplantation is a curative treatment for a combined immunodeficiency phenotype and might reduce the long-term risk of developing lymphoid malignancy. It has been considered in some patients with varying results. High mortality has been reported as conditioning regimens are poorly tolerated in these highly susceptible patients (1, 9).

## Conclusions

We presented the clinical and immunologic manifestations of three unrelated patients with different phenotypes of ligase IV deficiency: a classical presentation and two unusual presentations resembling CVID. We also report a novel genetic variant paired with a known pathogenic mutation in heterozygous compound state in a patient with classic phenotype of the ligase IV syndrome.

In the last few years, the spectrum of clinical manifestations caused by ligase IV deficiency has widened, making it very difficult to establish an accurate clinical diagnosis. So, although the *LIG4* syndrome diagnosis should be considered in the presence of characteristic stigmata, today, we know that the diagnostic suspicion must go beyond these features since many patients can be asymptomatic or present with a mild clinical disease. Lymphopenia with diminished B cells and a reduced proportion of naïve T cells represent important laboratory biomarkers that should prompt a consideration of *LIG4* syndrome in yet asymptomatic patients. Although it is unclear what the best approach to these patients is, a presymptomatic identification of the ligase IV deficiency has important prognostic implications and impacts treatment decisions, in particular regarding DNA-damaging agents and exposure to ionizing radiation (11, 17).

The acknowledgment that LIG4-deficient patients can present very different clinical manifestations, in many cases overlapping with other diseases, makes the use of NGS essential for the proper diagnosis of this condition, thus allowing better prognosis and adequate family counseling.

## Ethics Statement

Ethical review and approval was not required for the study on human participants in accordance with the local legislation and institutional requirements. Written informed consent to participate in this study was provided by the participants’ legal guardian/next of kin. Written informed consent was obtained from the minor(s)’ legal guardian/next of kin for the publication of any potentially identifiable images or data included in this article.

## Author Contributions

ACC collected and synthesized the data, is responsible for literature review, and drafted the manuscript. RM and SB were the medical doctors of patients, participated in literature review, and revised the manuscript for intellectual content. JF performed genetic analysis. CM performed pathology analysis. CN revised the manuscript for intellectual content. AIC was the medical doctor of patients, performed bibliographical research, and revised the manuscript for intellectual content. JN was the medical doctor of patients, was responsible for literature review, and drafted the manuscript data. All authors contributed to the article and approved the submitted version.

## Funding

The present publication was funded by Fundação Ciência e Tecnologia, IP national support through CHRC (UIDP/04923/2020).

## Conflict of Interest

The authors declare that the research was conducted in the absence of any commercial or financial relationships that could be construed as a potential conflict of interest.

## Publisher’s Note

All claims expressed in this article are solely those of the authors and do not necessarily represent those of their affiliated organizations, or those of the publisher, the editors and the reviewers. Any product that may be evaluated in this article, or claim that may be made by its manufacturer, is not guaranteed or endorsed by the publisher.

## References

[1] Altmann T Gennery AR. DNA Ligase IV Syndrome; A Review. Orphanet J Rare Dis (2016) 11(1):1–7. doi: 10.1186/s13023-016-0520-1 27717373 PMC5055698

[2] Staines BA Chinn IK Alaez-Versón C Yamazaki-Nakashimada MA Carrillo-Sánchez K García-Cruz MLH. Failing to Make Ends Meet: The Broad Clinical Spectrum of DNA Ligase IV Deficiency. Case Series and Review of the Literature. Front Pediatr (2019) 6:426. doi: 10.3389/fped.2018.00426 30719430 PMC6348249

[3] Sharma R Lewis S Wlodarski MW. DNA Repair Syndromes and Cancer: Insights Into Genetics and Phenotype Patterns. Front Pediatr (2020) 8(October):1–17. doi: 10.3389/fped.2020.570084 33194896 PMC7644847

[4] Slatter MA Gennery AR. Update on DNA-Double Strand Break Repair Defects in Combined Primary Immunodeficiency. Curr Allergy Asthma Rep (2020) 20(10):57. doi: 10.1007/s11882-020-00955-z 32648006 PMC7347510

[5] Scully R Panday A Elango R Willis NA. DNA Double-Strand Break Repair-Pathway Choice in Somatic Mammalian Cells. Nat Rev Mol Cell Biol (2019) 20(11):698–714. doi: 10.1038/s41580-019-0152-0 31263220 PMC7315405

[6] Felgentreff K Baxi SN Lee YN Dobbs K Henderson LA Csomos K. Ligase-4 Deficiency Causes Distinctive Immune Abnormalities in Asymptomatic Individuals. J Clin Immunol (2016) 36(4):341–53. doi: 10.1007/s10875-016-0266-5 PMC4842108 27063650

[7] Rucci F Notarangelo LD Fazeli A Patrizi L Hickernell T Paganini T. Homozygous DNA Ligase IV R278H Mutation in Mice Leads to Leaky SCID and Represents a Model for Human LIG4 Syndrome. Proc Natl Acad Sci USA (2010) 107(7):3024–9. doi: 10.1073/pnas.0914865107 PMC2840307 20133615

[8] Tomkinson AE Naila T Bhandari SK. Altered DNA Ligase Activity in Human Disease. Mutagenesis (2020) 35(1):51–60. doi: 10.1093/mutage/gez026 31630206 PMC7317150

[9] Schober S Schilbach K Doering M Cabanillas Stanchi KM Holzer U Kasteleiner P. Correction to: Allogeneic Hematopoietic Stem Cell Transplantation in Two Brothers With DNA Ligase IV Deficiency: A Case Report and Review of the Literature. BMC Pediatr (2019) 19:346. doi: 10.1186/s12887-019-1724-z 31604460 PMC6788020

[10] Plowman PN Bridges BA Arlett CF Hinney A Kingston JE. An Instance of Clinical Radiation Morbidity and Cellular Radiosensitivity, Not Associated With Ataxia-Telangiectasia. Br J Radiol (1990) 63(752):624–8. doi: 10.1259/0007-1285-63-752-624 2400879

[11] Riballo E Critchlow SE Teo SH Doherty AJ Priestley A Broughton B. Identification of a Defect in DNA Ligase IV in a Radiosensitive Leukaemia Patient. Curr Biol (1999) 9(13):699–702. doi: 10.1016/S0960-9822(99)80311-X 10395545

[12] Brunet BA Dave N. Unique Heterozygous Presentation in an Infant With DNA Ligase IV Syndrome. Ann Allergy Asthma Immunol (2017) 119(4):379–80. doi: 10.1016/j.anai.2017.07.017 28866308

[13] Sun B Chen Q Wang Y Liu D Hou J Wang W. LIG4 Syndrome: Clinical and Molecular Characterization in a Chinese Cohort. Orphanet J Rare Dis (2020) 15(1):1–9. doi: 10.1186/s13023-020-01411-x 32471509 PMC7257218

[14] Luo X Liu Q Jiang J Tang W Ding Y Zhou L. Characterization of a Cohort of Patients With LIG4 Deficiency Reveals the Founder Effect of P.R278L, Unique to the Chinese Population. Front Immunol (2021) 12. doi: 10.3389/fimmu.2021.695993 PMC8498043 34630384

[15] Matsumoto K Hoshino A Nishimura A Kato T Mori Y Shimomura M. DNA Ligase IV Deficiency Identified by Chance Following Vaccine-Derived Rubella Virus Infection. J Clin Immunol (2020) 40(8):1187–90. doi: 10.1007/s10875-020-00831-5 32914283

[16] Gerasimou P Koumas L Miltiadous A Kyprianou I Chi J Gavrielidou R. The Rare DNA Ligase IV Syndrome: A Case Report. Hum Pathol Case Rep (2020) 22:200442. doi: 10.1016/j.ehpc.2020.200442

[17] Chadha P Thibodeau R Jafroodifar A Majmudar A. A Case Report of an Adolescent With Ligase-4 Deficiency and the Potential Dangers of Ionizing Radiation in This Rare Patient Population. Radiol Case Rep (2021) 16(10):2890–3. doi: 10.1016/j.radcr.2021.07.002 PMC8349749 34401020

[18] Murray JE Bicknell LS Yigit G Duker AL van Kogelenberg M Haghayegh S. Extreme Growth Failure Is a Common Presentation of Ligase IV Deficiency. Hum Mutat (2014) 35(1):76–85. doi: 10.1002/humu.22461 24123394 PMC3995017

[19] Tangye SG Al-herz W Bousfiha A Chatila T Cunningham-rundles C Etzioni A. Human Inborn Errors of Immunity: 2019 Update on the Classification From the International Union of Immunological Societies Expert Committee. J Clin Immunol (2020) 40(1):24–64. doi: 10.1007/s10875-019-00737-x 31953710 PMC7082301

[20] Ijspeert H Warris A van der Flier M Reisli I Keles S Chishimba S. Clinical Spectrum of LIG4 Deficiency Is Broadened With Severe Dysmaturity, Primordial Dwarfism, and Neurological Abnormalities. Hum Mutat (2013) 34(12):1611–4. doi: 10.1002/humu.22436 PMC3910166 24027040

[21] Tilgner K Neganova I Moreno-Gimeno I Al-Aama JY Burks D Yung S. A Human iPSC Model of Ligase IV Deficiency Reveals an Important Role for NHEJ-Mediated-DSB Repair in the Survival and Genomic Stability of Induced Pluripotent Stem Cells and Emerging Haematopoietic Progenitors. Cell Death Differ (2013) 20(8):1089–100. doi: 10.1038/cdd.2013.44 PMC3705601 23722522

[22] Madhu R Beaman GM Chandler KE O’Sullivan J Urquhart JE Khan N. Ligase IV Syndrome can Present With Microcephaly and Radial Ray Anomalies Similar to Fanconi Anaemia Plus Fatal Kidney Malformations. Eur J Med Genet (2020) 63(9):103974. doi: 10.1016/j.ejmg.2020.103974 32534991 PMC7445424

[23] Chistiakov DA Voronova NV Chistiakov AP. Ligase IV Syndrome. Eur J Med Genet (2009) 52(6):373–8. doi: 10.1016/j.ejmg.2009.05.009 19467349

[24] O’Driscoll M Cerosaletti KM Girard P Dai Y Stumm M Kysela B. DNA Ligase IV Mutations Identified in Patients Exhibiting Developmental Delay and Immunodeficiency. Mol Cell (2001) 8(D):1175–85. doi: 10.1016/S1097-2765(01)00408-7 11779494

[25] Riballo E Doherty AJ Dai Y Stiff T Oettinger MA Jeggo PA. Cellular and Biochemical Impact of a Mutation in DNA Ligase IV Conferring Clinical Radiosensitivity. J Biol Chem (2001) 276(33):31124–32. doi: 10.1074/jbc.M103866200 11349135

[26] Girard PM Kysela B Härer CJ Doherty AJ Jeggo PA. Analysis of DNA Ligase IV Mutations Found in LIG4 Syndrome Patients: The Impact of Two Linked Polymorphisms. Hum Mol Genet (2004) 13(20):2369–76. doi: 10.1093/hmg/ddh274 15333585

